# Implementation and Evaluation of a Dynamic Neck Brace Rehabilitation Device Prototype

**DOI:** 10.1155/2022/6887839

**Published:** 2022-10-27

**Authors:** Mostafa El-Hussien Ibrahem, Mohamed Tarek El-Wakad, Mostafa Saied El-Mohandes, Sherif A. Sami

**Affiliations:** ^1^Biomedical and Systems Engineering Department, Higher Institute of Engineering, El-Shorouk Academy, Cairo, Egypt; ^2^Biomedical Engineering Department, Faculty of Engineering, Helwan University, Cairo, Egypt; ^3^Faculty of Engineering and Technology, Future University in Egypt, Cairo, Egypt; ^4^Biomedical Engineering and Systems Department, Faculty of Engineering, Cairo University, Giza, Egypt

## Abstract

Rehabilitation assistive devices for head/neck pain treatment cannot allow dynamic changes in position and orientation of the head/neck. Moreover, such devices can neither be used simultaneously nor can they assess the patients' head/neck conditions. This paper aims at designing and implementing a novel dynamic head/neck brace that provides static and dynamic support and/or traction at symmetric and asymmetric positions. This device also provides assessments of the head/neck stiffness for the purpose of fulfilling diagnoses of the head/neck disorders. The device was used and evaluated for its range of motion and its symmetric traction capability using two control modalities. In addition, it was also evaluated in determining the stiffness of the head/neck throughout a simulating mechanical model involved in a set of springs. The device could apply right/left lateral bending to the head/neck ranged −6.97 ± 0.01° to 7.02 ± 0.01° with accuracies of 99.89% and 99.48%, and flexion/extension ranged −8.10 ± 0.02° to 8.12 ± 0.01° with accuracies of 99.57% and 99.42%, respectively, throughout a traction phase of 20 mm. The practical measurements through the symmetric traction tests showed some deviations as compared to that being calculated. Such deviations were greater in flexion/extension rather than the right/left lateral bending. The mean of the obtained error was less than 0.34° for all situations of tests. The accuracies of stiffness measurement of the mechanical model were 99.78% and 99.96%, respectively, throughout performing stair and step tests. The paper presented a novel design of a dynamic head/neck brace that provides support and/or traction to any head/neck positions and capable of evaluating the head/neck stiffness during cervical traction.

## 1. Introduction

Neck pain is one of the most reported complaints of the musculoskeletal system, and its point prevalence varies between 10% and 22%, respectively, depending on the population and the definition of neck pain. It is estimated that 20% to 70% of the adult population will experience neck pain in their lifetime [1–7]. Nonsurgical treatment is the first step to recovery. Treatment and reduction of neck pain depend on a set of treatment protocols, which depends on different techniques, including medication, rest, massage, home exercises, ultrasound, hydrotherapy, electrotherapy, chiropractic care, and physical therapy [8, 9]. The physical therapy programs rely on the mechanical and electrical medical devices such as cervical traction devices, muscle stimulation and strengthening devices, and neck braces [8, 10].

Cervical traction pulls the head away from the neck to unload the components of the spine by stretching ligaments, muscles, and functional spinal units, which will decrease intradiscal pressure, thereby relieving symptoms. Traction is applied manually or mechanically, the tension may be intermittent or continuous, and the patient may be upright or supine when tension is applied [8]. Studies on cervical traction have different aims: comparing the effect of traction on the myoelectric activity [11, 12], examining traction therapy efficacy in chronic neck pain [13], developing a clinical prediction rule (CPR) to identify patients with neck pain likely to improve with cervical traction [14], exploring the effect of the continuous and intermittent traction on the treatment of cervical radiculopathy with infrared radiation [15], and examining the effectiveness of cervical traction besides exercise [16]. The value of cervical traction treatment has been often questioned because studies on its usefulness have generally been inconclusive. Some studies reported improvements in patients treated with cervical traction [15–17]. Meanwhile, other studies showed that cervical traction is not effective in the treatment [11–13].

Neck braces, which are used in the first place to stabilize the neck vertebrae and reduce pressure on the cervical vertebrae, thus reducing the pain that the patient feels [8, 10]. Many research studies on developing new designs of the neck brace have been reported. In an attempt to improve quality of life for amyotrophic lateral sclerosis (ALS) patients, a static brace was designed by the Houston Methodist Hospital to improve balance, breathing, and mobility by supporting the head posteriorly with no restriction of the chest or mandible area [18]. Another cervical orthotic static brace (known as Sheffield Support Snood) was designed for people who were affected by progressive neck muscle weakness [19–22]. Although, some commercial neck braces were recently used as cervical traction devices; however, they were passive and do not provide any active dynamic motion [23–25].

Parallel robot configuration has been used in a variety of practical applications such as microrobot [26–30], vehicle and aircraft simulators [31–35], and medical devices [36–43]. In medical fields, particularly orthostatic devices, the development of an active parallel manipulators is still in its early stages; however, they have a promising future. Such active wearable devices can propose a new approach to rehabilitation of patients.

The different neck brace designs based on the parallel robot configuration were implemented. One of them, which was dynamic, was used as a measuring tool for human head movement [44]; then, it was developed as an assistive device for dropped head syndrome (DHS) [45]. Another one was developed as a special wearable therapy device for assisting patients who were suffering from the head/neck posture problems [46]. A preliminary design for a dynamic neck brace was recently developed. It could be used to support the neck in different positions as well as providing cervical traction [1].

Physical therapy of head/neck pain treatments of patients are recently improved by using different designs of assistive devices; however, these devices are limited at providing only fixed protocols of treatments which does not involve dynamic change in the position and orientation of the head/neck. In addition, limited to the inability to be used simultaneously, and they cannot be used to assess patients with the head/neck conditions.

Nonetheless, it is possible to further improve the efficacy by combining different treatment methods simultaneously. With this goal, this research study introduces a prototype of dynamic neck brace that allows measuring the position and orientation of the head as well as the pressure forces on the head due to the traction effect. The measurements of position and orientation are based on the outcome readings from three linear actuators in the device. By using this device, the biomechanical parameters relevant to the head/neck stiffness can be assessed and then diagnosed. The device allows applying different protocols of treatment. It allows support and traction to the head/neck in symmetric and asymmetric positions. The traction can be controlled to be performed statically or dynamically with different rates based on the protocol of the movement required.

## 2. Materials and Methods

A kinematic analysis, the CAD model design, and motion analysis of this brace were previously elaborated where the workspace and range of motion of the design were computed, and the CAD model design of the brace was validated [1]. In this paper, key characteristics of the prototype, the control interface, measuring stiffness of different springs that mimic the human neck parameter, and evaluation of the system are clarified.

### 2.1. Kinematic and CAD Model Design

**Figure 1:**
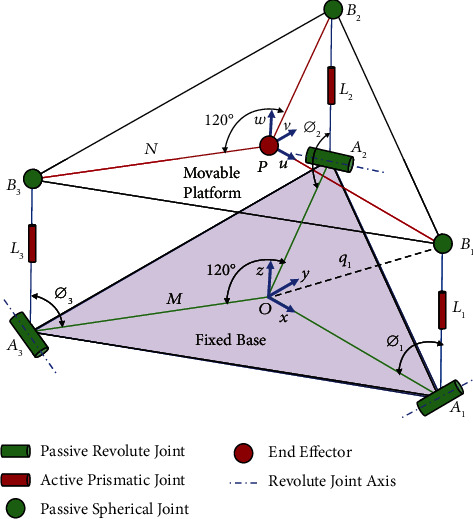
Kinematic diagram of 3RPS parallel manipulator [1].

**Figure 2:**
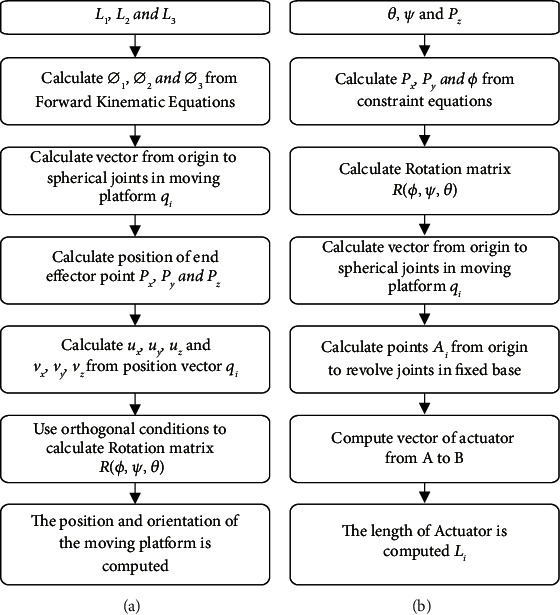
(a) Forward and (b) inverse kinematic flow diagram.

**Figure 3:**
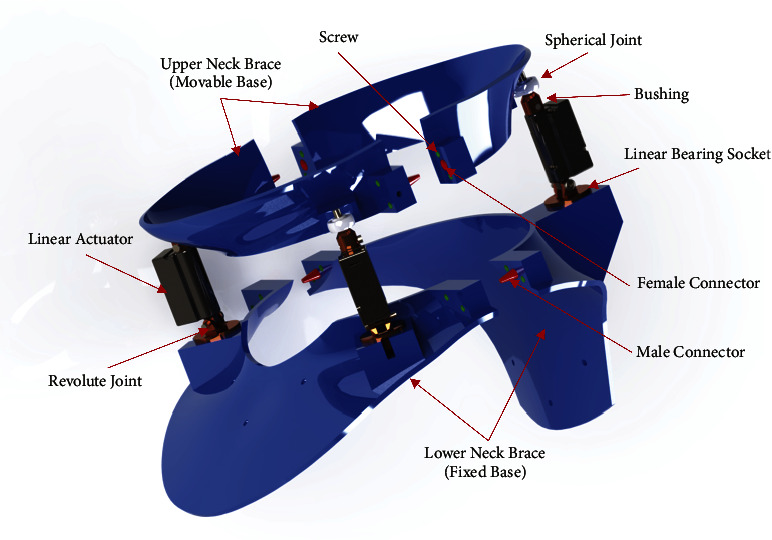
Neck brace CAD model [1].

The brace was designed based on the three revolute-prismatic-spherical (3RPS) parallel configurations, which consists of a fixed base connected to the three identical limbs with a movable platform. Each limb consists of a revolving, prismatic, and spherical joint. A kinematic diagram for the device was prepared to facilitate calculating the forward and inverse kinematic equations (Figures 1 and 2). A software program was created using MATLAB to validate the forward and inverse kinematic equations and to obtain workspace and range of motion. In addition, a CAD designed model was performed using SolidWorks (Figure 3). The design includes a fixed base and a movable platform of a radius 95 mm. Each platform consists of two parts connected with screws and interconnected parts with male and female connectors to increase the rigidity. The three actuating limbs of 53 mm length of each, each limb can stretch by up to 20 mm. A motion analysis to the movability of the device was performed to validate its movements versus the analytical calculations.

### 2.2. Hardware Configurations of the Neck Brace Prototype

**Figure 4:**
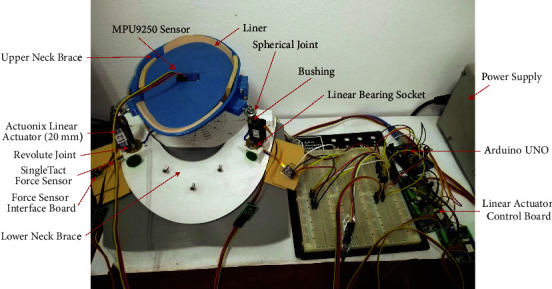
Neck brace prototype.

The fixed and movable bases of the neck brace prototype shown in Figure 4 were made using semirigid 3 mm fused deposition modelling (FDM) PLA, separated by a 6 mm gap in the neutral position. The movable base of the neck brace movements can be achieved by three mechanical linear actuators (Actuonix PQ12-100-12-P).

Each actuator has a length of 48 mm with a controlled stroke up to 20 mm. The actuator can sustain a load up to 50 N at operating speed up to 10 mm/s at no load. Each actuator has a revolving joint at the base and a spherical joint at the top. The actuator is connected to the spherical joint with bushing. The revolute joint was fabricated as linear bearing with a revolving shaft and a socket. At the base of the revolute joint, a calibrated force sensor (SingleTact, CS15-450N) is mounted. The brace is lined with Plastazote polyethylene foam. Arduino Uno was used to control the three actuators and read the data from the actuator's position feedback and force sensor. The linear actuator is driven at 12 V using the Actuonix Linear Actuator Control Board. Gyroscope sensor (MPU9250) was used to obtain the orientation of the movable platform.

### 2.3. Control of the 3RPS Parallel Manipulator

**Figure 5:**
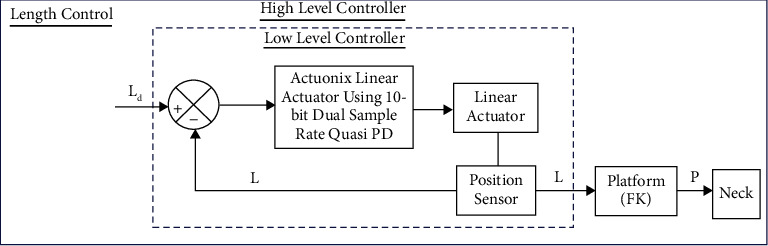
Length control of the system.

**Figure 6:**
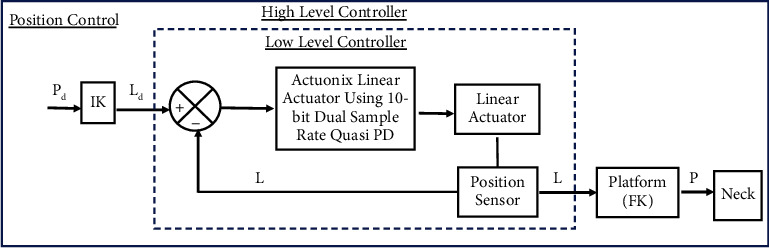
Position control of the system.

Two control modalities: length and position control were implemented at the joint space. Sensors on each limb give a real-time joint position to the controller that allows closed-loop control using the Actuonix Linear Actuator Control Board, which is a stand-alone closed-loop control board specifically designed for Actuonix actuators. Actuonix motor control (IC) uses a software-based algorithm to optimize the position and speed control. It uses a 10-bit dual sample rate Quasi PD controller. Since it is difficult to detect the motion of the parallel platform directly, the length and position controller were designed in the joint space based on the position feedback of the actuator. The control topology for length and position protocol control is shown in Figures 5 and 6 respectively. The control topology consists of a high-level controller and a low-level controller.

The high-level controller uses the actuated input *L*_d_, where *L*_*d*_=[*L*_1_, *L*_2_, *L*_3_] denotes the desired position of the actuator in the joint space at length control modality, while at position control modality the high-level controller maps the desired motion of the platform in Cartesian space (*P*_d_) into the joint space variable using inverse kinematics (*L*_d_). Where *P*_*d*_=[*P*_*x*_, *P*_*y*_, *P*_*z*_, *R*_*x*_, *R*_*y*_, *R*_*z*_]^*T*^ is the vector of pose variables of the moving platform and *L*_*d*_=[*L*_1_, *L*_2_, *L*_3_] is the vector of the actuated joint. Part of the high-level controller also computes the Cartesian position vectors of the platform (P) using the joint position feedback via forward kinematics, where P is the desired motion of the robotic brace in the workspace.

The low-level controller, for both the length and position control modality are the same and consisting of individual PD controllers for each joint, receives the desired length position from the high-level controller, and performs the closed-loop control on the joint position. The error between the desired position and the actual position (*L*) is the input signal of the PD controller that provides the driving forces to drive each electric actuator.

### 2.4. Range of Motion and Forward Kinematic Validation

The mobility of the movable platform was validated vs its kinematic calculations. The flexion/extension and right/left lateral bending movements were subjected to validation tests. For validating the flexion/extension movement, all actuators were activated to increase their lengths in steps of 2 mm each, up to their full stroke (Table 1). While for validating the right/left lateral bending movements, the actuator *L*_1_ was activated to increase its length in steps of 1 mm only, up to its half full stroke and the other two actuators *L*_2_ and *L*_3_ were activated simultaneously to increase their lengths by 2 mm up to its full stroke (Table 1).

All increases in length for all the actuators were synchronized to be performed simultaneously and kept for 20 sec intervals. The test was repeated in the opposite direction where the actuator was activated to decrease its length by the same manner to complete a cycle. The cycles of tests were repeated five times, and the motion was recorded through potentiometers on the actuators as well as a gyroscope sensor. The position and orientation of the movable platform were determined mathematically based on the data from the actuator feedback position in the joint space. The gyroscope sensor (MPU9250) was used to measure the orientation of the movable platform. The mean and standard deviation of each cycle was determined. The error between the feedback measurements from the actuator and the corresponding calculated values was determined.

### 2.5. Symmetric Traction and Inverse Kinematic Validation

The system was also validated for symmetric traction using position control modality in the joint space based on the position feedback of the actuator. The desired position and orientation of the end effector applied to the system and the length of the three actuators were calculated from the inverse kinematic equations. The angle changed from −7° to 7° about the *y*-axis (*θ*) (flexion/extension) Table 2, and from −6° to 6° about the *x*-axis (*ψ*) (lateral bending) Table 3. Each test was applied with an increment of 1° as intermittent traction for 5 cycles; each cycle contained push and relax phases for 20 sec.

The motion was recorded through potentiometers on the actuators as well as a gyroscope sensor. The position and orientation of the movable platform were determined mathematically based on the data from the actuator feedback position in the joint space. The gyroscope sensor (MPU9250) was used to measure the orientation of the movable platform.

### 2.6. Measurement of Stiffness Values

Four springs were first tested using different weights to get the stiffness of spring *K*. Each test was applied with loading and unloading the weights. The displacement values were measured, and the slope of the force-displacement curve was used to calculate the stiffness. The stiffness of the springs in each experiment, loading and unloading was calculated, and the average stiffness of each spring was then calculated.

**Figure 7:**
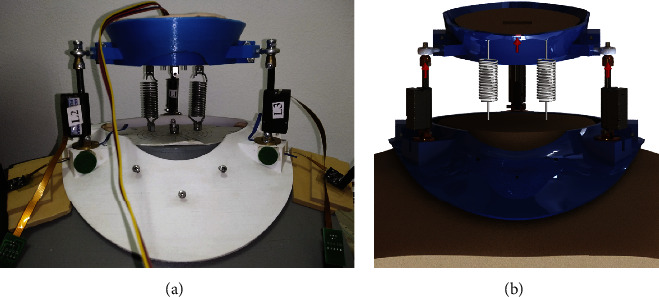
Exp. 1 setup where spring 3 located at *L*_3_ Ext and spring 4 located at *L*_2_ Ext (a) practical and (b) SolidWorks simulation.

A mechanical model consists of different springs connected in parallel was used to mimic the behavior of human neck and four experiments were carried out using four springs (Table 4). Experiment 1 (Exp 1) was carried out with the spring located at *L*_2_ Ext and *L*_3_ Ext (Figure 7(a)), where Ext refer to the extension line of actuator. Experiment 2 (Exp 2) was carried out with the spring located at *L*_1_, *L*_2_ Ext, and *L*_3_ Ext. Experiment 3 (Exp 3) was carried out with the spring located at the origin point, *L*_2_ Ext, and *L*_3_ Ext. Experiment 4 (Exp 4) was carried out with the spring located at the origin point, *L*_1_, *L*_2_ Ext, and *L*_3_ Ext, where Ext refers to the extension line of the actuator position with the origin.

Each experiment was carried out using displacement input to the three actuators with two different modes of input as a stair input and as a step input. The stair input was applied from 0 mm to 20 mm with a 4 mm increment in the *z*-axis direction of the fixed base coordinating system with 20 sec for each step. The step input was applied with a step value of 20 mm and was applied as intermittent traction with a pull period of 40 sec and a relax period of 20 sec for two cycles. The step input used for Exp 1 was 20 mm, Exp 2 and Exp 3 were 16 mm, and Exp 4 was 10 mm. The time (t), position feedback (*L*_1_, *L*_2_, and *L*_3_), and forces (F_1_, F_2_, and F_3_) were measured. The relation between the force (*F*_T_) and displacement (*P*_Z_) was used to compute the stiffness (K) of the spring and each experiment was carried out three times and the mean stiffness was calculated.

In the stair input, the average displacement and average force for each step were calculated for 10 sec. The force-displacement curve was drawn using these data, and the stiffness was then calculated as the slope of the curve. In the step input, the average displacement and the average force were calculated for the two cycles and the average stiffness of the springs was calculated by dividing the average force by the average displacement. For cycle 1, the average displacement and average force were from 30 to 50 sec, respectively, while for cycle 2, they were from 90 to 110 sec, respectively.

The force values were validated using SolidWorks, where the same experiment setup was applied (Figure 7(b)). The input data were applied as stair and step inputs. The input to the actuators applied as the measured linear actuator feedback, and the force of each motor was recorded and compared to the experiment force measured by the force sensor.

## 3. Results

### 3.1. Range of Motion and Forward Kinematic Validation

The mobility of the movable platform was validated using the length control modality for the flexion/extension and right/left lateral bending movements. The desired length of the actuator applied to the system and the position and orientation of the movable platform were determined mathematically based on the data from the actuator feedback. Also, a gyroscope sensor (MPU9250) was used to measure the orientation of the movable platform.

The range of motion results showed that the system satisfying 3DOF with flexion/extension of −8.10 ± 0.02° to 8.12 ± 0.01° and accuracy of 99.57% and 99.42%, right/left lateral bending ranges from −6.97 ± 0.01° to 7.02 ± 0.01° with an accuracy of 99.89% and 99.48%, and maximum extension of the limbs ranges from 53 mm to 73 mm which allows fixing the neck in symmetric and asymmetric position. Using the MPU9250, for measuring the orientation of the platform, showed that the system satisfying flexion/extension with range of −8.14 ± 0.01° to 8.05 ± 0.02° with an accuracy of 99.03% and 99.64% and right/left lateral bending ranges from −6.95 ± 0.00° to 6.96 ± 0.02° with an accuracy of 99.56% and 99.68%, respectively.

**Figure 8:**
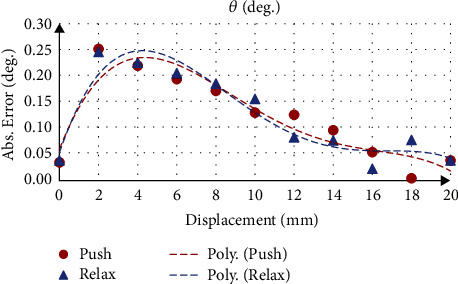
Movable platform orientation error using calculation.

**Figure 9:**
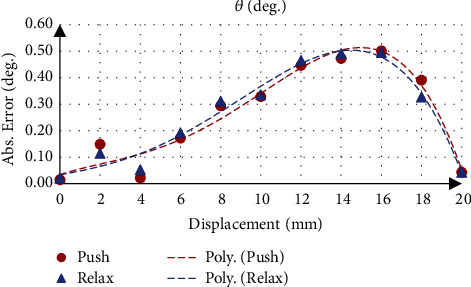
Movable platform orientation error using gyroscope sensor.

Taking flexion movement test as example, where actuator *L*_1_ varied and actuators *L*_2_ and *L*_3_ were constant. The position in the *z*-axis (*P*_Z_) changed from 0 to 6.66 mm, while the orientation about the *x*-axis (*ψ*) was 0° and about the *y*-axis (*θ*) changed from 0 to −8.06°. For the position *P*_Z_, the results show that the mean absolute error was 0.06 ± 0.04 mm, and the maximum position *P*_Z_ was 6.74 ± 0.01 mm with an accuracy of 98.76%. While the movable platform orientation about the *y*-axis (*θ*), the results show that the mean absolute error was 0.12 ± 0.08° (Figure 8) and the maximum flexion was −8.10 ± 0.02° with an accuracy of 99.57%. The gyroscope measurement results show that the mean absolute error was 0.26 ± 0.18° (Figure 9) and the maximum flexion was −8.14 ± 0.01° with an accuracy of 99.03%.

### 3.2. Symmetric Traction and Inverse Kinematic Validation

The system was validated for symmetric traction using position control modality in the joint space based on the position feedback of the actuator. The desired position and orientation of the end effector applied to the system and the length of the three actuators were determined from the inverse kinematic equations. The position and orientation of the movable platform were determined mathematically based on the data from the actuator feedback. Also, a gyroscope sensor (MPU9250) was used to measure the orientation of the movable platform.

Table 5 summarizes the calculated and measured orientation about the *y*-axis *θ* (Mean ± SD), which represents flexion/extension movement. The mean absolute error for all test trajectory in flexion movement direction was 0.13° using the calculation and 0.34° using the gyroscope measurements. Whereas, in extension movement, the direction was 0.09° using the calculation and 0.28° using the gyroscope measurements.

**Figure 10:**
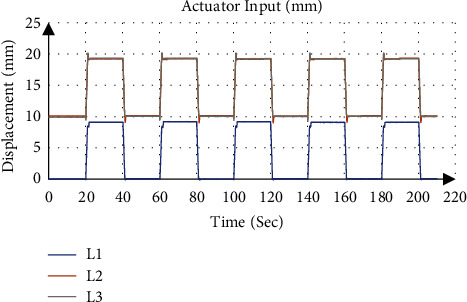
Actuator input at fixed angle 4° about *y*-axis.

**Figure 11:**
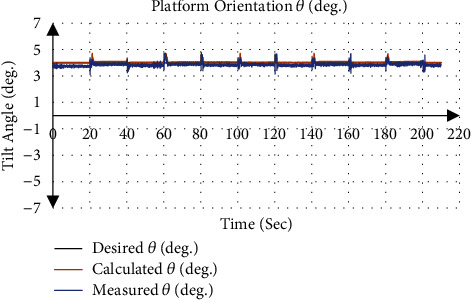
Orientation of end effector about *y*-axis *θ* at angle 4°.

Figure 10 gives an example of the actuator input calculated from the inverse kinematics and applied to the system, where the desired position and orientation was 7 mm for *P*_Z_, 0° for *ψ* and 4° for *θ* with applied traction of 9 mm.  Figure 11 shows that the calculated orientation about the *y*-axis *θ* was 4.07 ± 0.09° with an absolute error of 0.07°, while the gyroscope sensor measurement was 3.85 ± 0.11° with an absolute error of 0.15°.

Table 6 summarizes the calculated and measured orientation about the *x*-axis *ψ* (Mean ± SD), which represents right/left lateral bending movement. The mean absolute error for all test trajectory in the right lateral bending movement direction was 0.04° using the calculation and 0.15° using the gyroscope measurements. While in the left lateral bending movement, the direction was 0.06° using the calculation and 0.16° using the gyroscope measurements.

**Figure 12:**
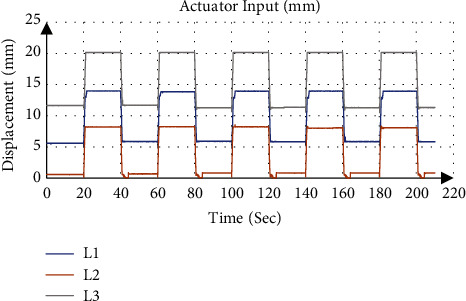
Actuator input at fixed angle −4° about x-axis.

**Figure 13:**
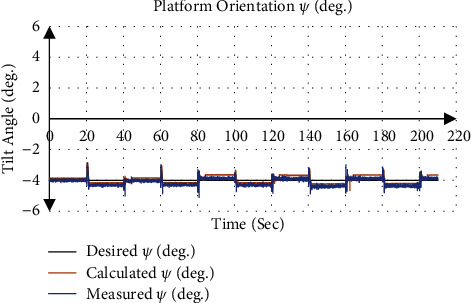
Orientation of end effector about *x*-axis *ψ* at angle −4°.

Figure 12 gives an example of the actuator input calculated from the inverse kinematics and applied to the system, where the desired position and orientation were 6 mm for *P*_Z_, −4° for *ψ*, and 0° for *θ* with applied traction of 8 mm.  Figure 13 shows that the calculated orientation about the *x*-axis *ψ* was −3.95 ± 0.24° with an absolute error of 0.05°, while the gyroscope sensor measurement was −4.11 ± 0.22° with an absolute error of 0.11°.

### 3.3. Measurement of Practical Stiffness Values

Results from testing spring 1 with different weights showed that the average stiffness of spring 1 was *K*_1_ = 1.24 ± 0.06 N/mm, while the average stiffness for spring 2 was *K*_2_ = 1.27 ± 0.08 N/mm, spring 3 was *K*_3_ = 1.27 ± 0.08 N/mm, and spring 4 was *K*_4_ = 1.27 ± 0.08 N/mm.

**Figure 14:**
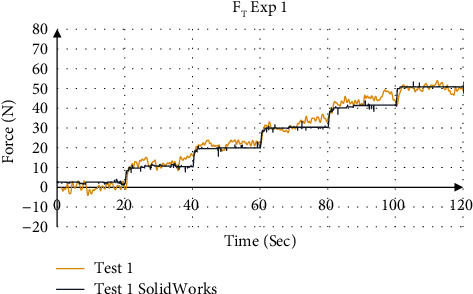
Force distribution using force sensors and SolidWorks simulation for Exp 1.

**Figure 15:**
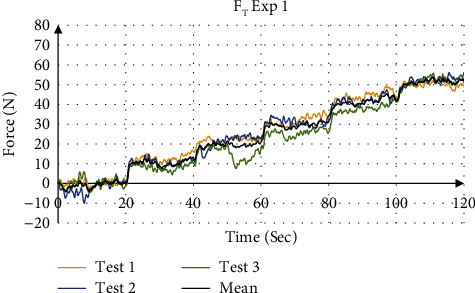
Total force measurement for Exp 1.

The validation of force values measured by the force sensors were compared to the simulation result from SolidWorks.  Figure 14 shows the force distribution of the measured force with the simulation results for Exp 1 (Test 1).  Figure 15 shows the mean total force distribution for each test and Table 7 summarizes the stiffness values calculated from the mean force displacement curve for each experiment. The four experiments showed that the device is able to measure the stiffness values using a stair input with an accuracy of 98.89% to 99.78%, respectively.

**Figure 16:**
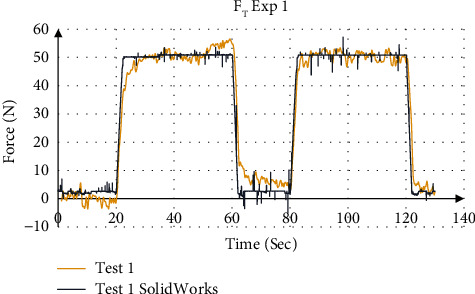
Force distribution using force sensors and SolidWorks simulation for Exp 1.

**Figure 17:**
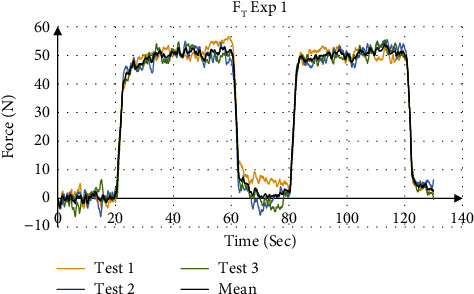
Total force measurement for Exp 1.

The validation of force values measured by the force sensors were compared to the simulation result from SolidWorks.  Figure 16 shows the force distribution of the measured force with the simulation results for Exp 1 (Test 1).  Figure 17 shows the mean total force distribution for each test and Table 8 summarizes the stiffness values calculated from the mean displacement and force for each experiment. The four experiments showed that the device is able to measure the stiffness values using a step input with an accuracy of 98.99% to 99.96%, respectively.

## 4. Discussion

The neck brace prototype was implemented and evaluated using two control modalities: length and position control. The proposed system was evaluated for the range of motion using the length control modality for flexion/extension and right/left lateral bending movement; the position control modality was used to evaluate the ability of the developed system to apply symmetric traction. The position and orientation of the movable platform were calculated from the linear actuator position feedback, and gyroscope sensor (MPU9250) was used to measure the orientation of the movable platform. The MPU sensor measurement showed no significant difference between the calculation and the direct measurement.

The developed system was able to apply flexion/extension with a range of −8.10 ± 0.02° to 8.12 ± 0.01° with accuracies of 99.57% and 99.42%, which limits motion to 12% of normal (−65° to 67°) [47]. The right/left lateral bending movement ranged from −6.97 ± 0.01° to 7.02 ± 0.01° with accuracies of 99.89% and 99.48%, which limits lateral bending motion to 17% of normal (−42° to 41°) [47]. Compared to Lingampally's study which had maximum angular tilt between −15° to 15° [46], as the design used in his study had maximum extension of the links ranging from 120 mm to 160 mm, our study used links ranging from 53 mm to 73 mm. Moreover, the fixed base and top platform radius were 320 mm and 250 mm, respectively, in his study, but our study used equal fixed and movable platforms with radius 95 mm.

In the symmetric traction test, the gyroscope measurement showed that the mean absolute error was greater than the calculated one, and the error was greater in flexion/extension movement than the right/left lateral bending. As the gyroscope measurement includes the errors from the linear actuators and the manufacturing parts include either the joints or the 3D printing parts, while the calculation method only includes the actuator errors. The maximum mean absolute error was less than 0.34° for all the tested trajectories and it was considered minor. This error could be from using the inverse kinematic equations, as the length of the actuators calculated from the equations and applied to the system is a fraction, not an integer number, causing a backlash in the actuators. However, as we mentioned, the error is still minor. Another cause could be from the accuracy of manufacturing the device parts either the 3D printing parts or the joint parts. In addition, the linear actuator mechanical backlash was 0.25 mm, and the sensitivity of the linear actuator was relatively low at the lower degrees compared to the higher degrees. This means that the device is not repeatable at very small degrees; however, these acute variations in the angle of traction have a little impact on cervical traction applications.

The developed system measured the stiffness of the different springs using two input modes: stair and step input. The stair input test showed a minimum accuracy of 98.89% for Exp 1 and a maximum accuracy of 99.78% for Exp 4, as the accuracy increased with increasing the stiffness of the springs. Whereas, the step input test showed that the minimum accuracy was 98.99% at Exp 4 and the maximum accuracy was 99.96% at Exp 1. This will allow for the assessment of the neck stiffness during the therapy protocol applied to the patient.

## 5. Conclusions

The paper presented a preliminary prototype for a novel dynamic neck brace, which combines the features of multifunction assistive device that provides controlling of both static and dynamic support and/or traction at any symmetric and asymmetric positions. Also, it can provide the assessment of the biomechanical parameters relevant to the head/neck stiffness for fulfilling diagnosis purposes of the head/neck disorders. These contributions bring critical insights for future development of dynamic neck braces and rehabilitation for patients suffering from neck pain. The future work of this paper is to perform tests on human subjects taking into consideration the speed as a control parameter and the wearability of the device.

## Figures and Tables

**Table 1:** Moving sequence of the actuators.

Movement	*Actuator input*		
	*L *_1_ (mm)	*L *_2_ (mm)	*L *_3_ (mm)
Flexion	On (20 mm)	Off (0 mm)	Off (0 mm)
Extension	Off (0 mm)	On (20 mm)	On (20 mm)
Right lateral bending	On (10 mm)	Off (0 mm)	On (20 mm)
Left lateral bending	On (10 mm)	On (20 mm)	Off (0 mm)

**Table 2:** Range of symmetric traction about *y*-axis (flexion/extension).

Movement	Traction (mm)	*P *_Z_ (mm)	*ψ* (deg)	*θ* (deg)
Flexion	2	6	0	−7
	5	5	0	−6
	6	5	0	−5
	9	4	0	−4
	12	3	0	−3
	14	2	0	−2
	17	1	0	−1

Extension	17	2	0	1
	14	4	0	2
	12	5	0	3
	9	7	0	4
	6	9	0	5
	5	10	0	6
	2	6	0	7

**Table 3:** Range of symmetric traction about *x*-axis (right/left lateral bending).

Movement	Traction (mm)	*P *_Z_ (mm)	*ψ* (deg)	*θ* (deg)
Right lateral bending	2	9	−6	0
	4	8	−5	0
	8	6	−4	0
	10	5	−3	0
	14	3	−2	0
	16	2	−1	0

Left lateral bending	16	2	1	0
	14	3	2	0
	10	5	3	0
	8	6	4	0
	4	8	5	0
	2	9	6	0

**Table 4:** Experiment setup and location of springs.

Exp	Spring	1	2	3	4
Exp 1	2 + 3		*L *_3_ ext	*L *_2_ ext	
Exp 2	2 + 3 + 4		*L *_3_ ext	*L *_2_ ext	*L *_1_
Exp 3	2 + 3 + 4		*L *_3_ ext	*L *_2_ ext	Origin
Exp 4	1 + 2 + 3 + 4	Origin	*L *_3_ ext	*L *_2_ ext	*L *_1_

**Table 5:** Symmetric traction about *y*-axis (flexion/extension).

Movement	Desired *θ* (deg)	Calculated *θ* (deg)	Measured *θ* (deg)
Flexion	−7	−6.99 ± 0.24	−7.80 ± 0.13
	−6	−5.85 ± 0.24	−6.59 ± 0.15
	−5	−5.08 ± 0.15	−5.07 ± 0.11
	−4	−3.95 ± 0.20	−4.39 ± 0.10
	−3	−2.86 ± 0.21	−3.21 ± 0.13
	−2	−1.78 ± 0.28	−2.08 ± 0.19
	−1	−0.71 ± 0.35	−0.78 ± 0.21

Extension	1	1.01 ± 0.22	0.48 ± 0.31
	2	2.04 ± 0.15	1.57 ± 0.23
	3	2.99 ± 0.16	2.71 ± 0.12
	4	4.07 ± 0.09	3.85 ± 0.11
	5	5.19 ± 0.09	5.11 ± 0.11
	6	6.10 ± 0.22	6.05 ± 0.14
	7	7.24 ± 0.11	7.39 ± 0.12

**Table 6:** Symmetric traction about *x*-axis (right/left lateral bending).

Movement	Desired *ψ* (deg)	Calculated *ψ* (deg)	Measured *ψ* (deg)
Right lateral bending	−6	−5.97 ± 0.11	−6.20 ± 0.11
	−5	−5.05 ± 0.09	−5.33 ± 0.11
	−4	−3.95 ± 0.24	−4.11 ± 0.22
	−3	−2.99 ± 0.15	−3.14 ± 0.22
	−2	−1.87 ± 0.26	−1.88 ± 0.27
	−1	−1.00 ± 0.13	−1.02 ± 0.13

Left lateral bending	1	0.99 ± 0.17	1.03 ± 0.19
	2	1.82 ± 0.20	1.78 ± 0.25
	3	2.98 ± 0.11	3.09 ± 0.15
	4	3.90 ± 0.16	4.00 ± 0.21
	5	5.04 ± 0.07	5.27 ± 0.11
	6	5.99 ± 0.15	6.33 ± 0.15

**Table 7:** Mean stiffness values using stair input.

Exp	K (N/mm)	Accuracy (%)
Exp 1	2.57 ± 0.02	98.89
Exp 2	3.78 ± 0.16	99.08
Exp 3	3.82 ± 0.08	99.70
Exp 4	5.06 ± 0.11	99.78

**Table 8:** Mean stiffness values using step input.

Exp	K (N/mm)	Accuracy (%)
Exp 1	2.54 ± 0.03	99.96
Exp 2	3.82 ± 0.10	99.73
Exp 3	3.81 ± 0.09	99.94
Exp 4	5.10 ± 0.10	98.99

## Data Availability

The data used to support the findings of this study are included within the article.
